# Cardiovascular and cancer mortality in Brazil from 1990 to 2017

**DOI:** 10.1590/1516-3180.2019.1372160319

**Published:** 2019-07-15

**Authors:** Paulo Andrade Lotufo

**Affiliations:** I MD, DrPH. Full Professor, Department of Internal Medicine, Faculdade de Medicina da Universidade de São Paulo (FMUSP), São Paulo (SP), Brazil.

**Figure 1.**
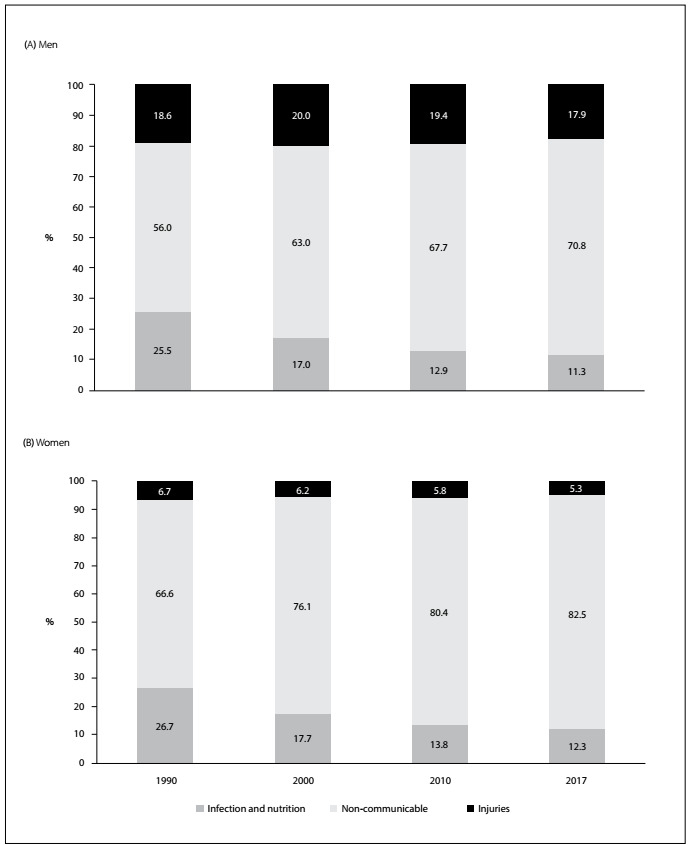
Evolution of proportional mortality relating to all causes for men (A) and women (B), due to infectious and nutritional diseases, non-communicable disorders and injuries in Brazil.

The profile of mortality in Brazil has changed over the last three decades. Deaths due to infections, nutritional diseases and maternal causes accounted for 25% of all occurrences in 1990. In 2017, they represented approximately 10%. Injuries are now the cause in almost 20% of deaths among men and 5% among women. Hence, non-communicable diseases are proportionally increasing as the cause of death for both sexes (Figure 1). This nosological category encompasses cardiovascular, respiratory, digestive, neurological and renal diseases, along with cancer. The most frequent components are cardiovascular diseases and cancer, both worldwide and in Brazil.^1^^-^^2^

Recently, an analysis conducted at the Center for Diseases Control and Prevention revealed that cancer may be about to surpass heart diseases as the leading killer in the United States.^3^ I analyzed the trends of both cancer and cardiovascular diseases (including heart and cerebrovascular diseases) in Brazil using data from the Global Burden of Diseases study 2017, which are available online (http://ghdx.healthdata.org/gbd-results-tool). This description follows three steps: first, the total number of deaths and the proportional mortality; second, rates according to the population each year; and third, age-standardized rates.

**Figure 2.**
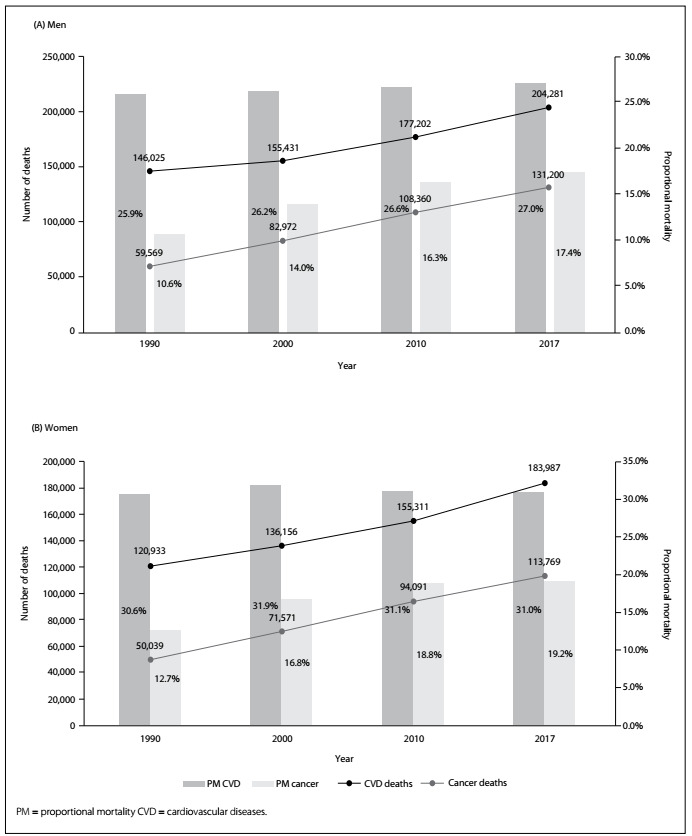
Evolution of number of deaths and proportional mortality relating to all causes for men (A) and women (B), due to cardiovascular diseases and cancer in Brazil.

Figure 2 displays four moments over these decades (1990-2017). It shows that the number of deaths due to cardiovascular diseases was higher than the number due to cancer, but that a significant change took place over this period. In 1990, the number of deaths due to circulatory disorders was 140% higher than the number due to cancer; but in 2016, the number due to cardiovascular diseases was 60% greater than the number due to cancer. The proportional mortality due to circulatory disorders remained unchanged, but it increased significantly for death due to cancer. Among men, the proportional mortality increased from 10.6% (1990) to 17.4% (a relative increase of 65%). Among women, the proportional mortality increased from 12.7% (1990) to 19.2% (a relative increase of 50%).

**Figure 3.**
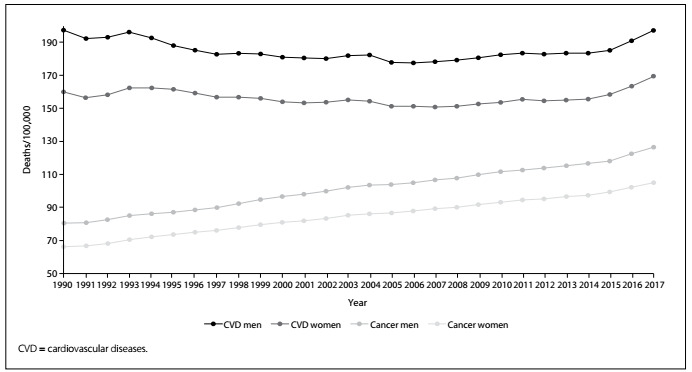
Crude death rates due to cardiovascular diseases and cancer in Brazil from 1990 to 2017.

**Figure 4.**
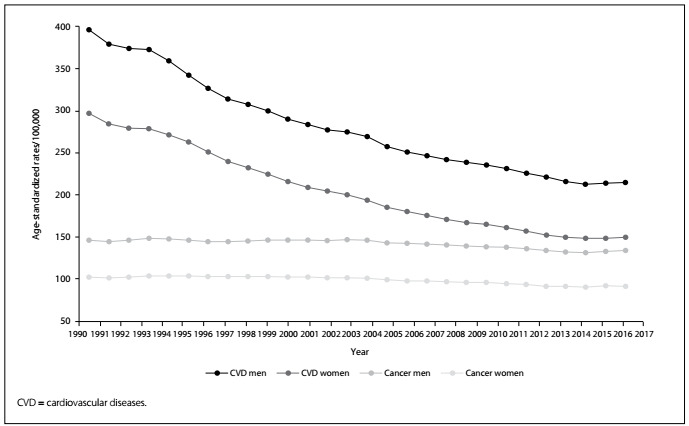
Age-standardized death rates due to cardiovascular diseases and cancer in Brazil from 1990 to 2017.

Figure 3 shows the trends in the numbers of deaths divided by the population of each year (crude rates). Visually, it is possible to speculate that cardiovascular rates are declining or flattening; in contrast, the rates due to cancer increased monotonically during this period. After adjustment for the difference in age strata over this period, as shown in Figure 4, it is easy to understand that the patterns for the risks of death due to circulatory disorders and cancer are different. The decline in age-standardized death rates due to cardiovascular diseases is steeper than that of the cancer rates. Table 1 presents the annual percentage change in the age-standardized death rates and shows that the decline in circulatory diseases over the period from 1990 to 2017 occurred at a faster pace. However, for both categories and both sexes, the rate of declining slowed down over the last five years of observation (2013-2017).

**Table 1.** Annual percentage change in age-standardized death rates due to cardiovascular diseases and cancer in Brazil from 1990 to 2017, and over the last ten years (2008-17) and last five years (2013-17) of observation

	Cardiovascular diseases		Cancer	
	Men	Women	Men	Women
1990-2017	-2.26	-2.55	-0.32	-0.41
Last 10 years	-1.32	-1.46	-0.53	-0.57
Last 5 years	-0.71	-0.52	-0.10	-0.17

Concluding, in contrast to what has been described in the United States, cancer deaths in Brazil are not surpassing fatal cases due to cardiovascular diseases. In this country, the number of deaths due to cardiovascular diseases and the risk of death due to these diseases, independent of aging, are higher than the numbers and risks relating to cancer, for both sexes. A more detailed explanation according to the types of circulatory disorders and types of cancer will be presented in forthcoming issues of the *São Paulo Medical Journal*.
